# The Protective Role of L-Cysteine in the Regulation of Blood–Testis Barrier Functions—A Brief Review

**DOI:** 10.3390/genes15091201

**Published:** 2024-09-12

**Authors:** Jeffrey Justin Margret, Sushil K. Jain

**Affiliations:** Department of Pediatrics, Louisiana State University Health Sciences Center, Shreveport, LA 71103, USA; jeffrey.justinmargret@lsuhs.edu

**Keywords:** blood–testis barrier, claudins, glutathione, L-cysteine, male infertility, oxidative stress, spermatogenesis, testosterone, tight junction

## Abstract

Blood–testis barrier (BTB) genes are crucial for the cellular mechanisms of spermatogenesis as they protect against detrimental cytotoxic agents, chemicals, and pathogens, thereby maintaining a sterile environment necessary for sperm development. BTB proteins predominantly consist of extensive tight and gap junctions formed between Sertoli cells. These junctions form a crucial immunological barrier restricting the intercellular movement of substances and molecules within the adluminal compartment. Epithelial tight junctions are complex membrane structures composed of various integral membrane proteins, including claudins, zonula occludens-1, and occludin. Inter-testicular cell junction proteins undergo a constant process of degradation and renewal. In addition, the downregulation of genes crucial to the development and preservation of cell junctions could disrupt the functionality of the BTB, potentially leading to male infertility. Oxidative stress and inflammation may contribute to disrupted spermatogenesis, resulting in male infertility. L-cysteine is a precursor to glutathione, a crucial antioxidant that helps mitigate damage and inflammation resulting from oxidative stress. Preclinical research indicates that L-cysteine may offer protective benefits against testicular injury and promote the expression of BTB genes. This review emphasizes various BTB genes essential for preserving its structural integrity and facilitating spermatogenesis and male fertility. Furthermore, it consolidates various research findings suggesting that L-cysteine may promote the expression of BTB-associated genes, thereby aiding in the maintenance of testicular functions.

## 1. Introduction

The blood–testis barrier (BTB) is important in segregating the cellular processes of spermatogenesis by acting as a barrier against harmful cytotoxic drugs, chemicals, and microorganisms, thereby safeguarding the seminiferous tubules and ensuring a sterile environment crucial for the development of germ cells [1]. Spermatogenesis is modulated by various internal and external factors, receiving regulatory signals from multiple cell types within the testis [2]. Leydig cells and Sertoli cells are the primary cell types involved in the regulation of testicular functions. Leydig cells, situated in the interstitial space of the testes, play a crucial role in the production and release of androgens, which are vital for the masculinization of male embryos and the initiation and sustenance of spermatogenesis. In contrast, Sertoli cells, found within the seminiferous tubules, are integral to the regulation of spermatogenesis and provide essential nutrients to the germ cells [3].

The initiation of puberty activates the hypothalamic–pituitary–gonadal axis, leading to the secretion of luteinizing hormone (LH) and follicle-stimulating hormone (FSH). LH acts on the testes’ Leydig cells, promoting testosterone synthesis [4]. Leydig cells are the primary and most significant source of androgenic hormones in mammals [5]. Meanwhile, Sertoli cells are essential for the support and maintenance of germ cells by secreting a range of growth factors that supply essential nutrients for testicular development and the process of spermatogenesis [3]. Sertoli cells are specialized epithelial cells characterized by their polarization, extending from the base of the seminiferous tubule to its lumen. These cells project extensive cytoplasmic processes that interact with neighboring Sertoli cells and develop germ cells, contributing to the formation of specialized cell junctions within the seminiferous epithelium. In the interstitium, Leydig cells synthesize testosterone upon stimulation by LH. This hormone is essential in maintaining the BTB, facilitating spermatogenesis and fertility, and regulating the assembly and disassembly of junctions between Sertoli and germ cells [6]. This review highlights several genes associated with the BTB that play crucial roles in maintaining its structural integrity, as well as in supporting spermatogenesis and male fertility. Additionally, this review also summarizes the findings from various studies indicating that L-cysteine may enhance BTB-related gene expressions and contribute to the preservation of testicular functions.

## 2. Blood–Testis Barrier

The BTB plays a key role in spermatogenesis. It is situated at the base of the seminiferous tubule, effectively partitioning the epithelium into two separate compartments: basal and adluminal. The formation of the BTB occurs during the developmental period between 12 and 14 years of age in the testes of human males, while in rats, this barrier is established between 17 and 21 days after birth [7,8]. Its primary function is to isolate germ cells located in the adluminal compartment from the circulatory and lymphatic systems, while also facilitating local immune suppression, thereby creating an immunoprivileged microenvironment necessary for the successful progression of meiosis [6]. In the active phase of spermatogenesis, germ cells undergo differentiation and traverse the BTB. This process is distinctly dynamic, characterized by cell projections that facilitate contact with neighboring cells, while adhesion molecules are meticulously reorganized to allow the movement of germ cells without compromising the integrity of the barrier [9]. It serves as a physical barrier that segregates the cellular processes of spermatogenesis, which includes the development of germ cells such as spermatocytes, spermatids, and sperm. In addition, it acts as a barrier against harmful substances such as cytotoxic drugs, chemicals, and microorganisms, including bacteria, viruses, and fungi, thereby safeguarding the seminiferous tubules and ensuring a sterile environment essential for the development of germ cells [1].

Within the seminiferous epithelium, the development of germ cells behind the BTB necessitates a continuous cycle of degradation and renewal of inter-testicular cell junctions. This dynamic process facilitates the translocation of immature germ cells from the basal lamina to the adluminal compartment during spermatogenesis, followed by the eventual release of mature spermatids into the tubular lumen during spermiation. Additionally, the removal of cellular debris from the epithelium is essential throughout spermatogenesis, suggesting the involvement of proteases, protease inhibitors, and components of cell junctions in these processes [10]. Furthermore, testosterone produced by Leydig cells, stimulated by luteinizing hormone, is crucial for sustaining the BTB, supporting spermatogenesis, and ensuring fertility while also influencing the assembly and disassembly of Sertoli–germ cell junctions [6].

## 3. Structural Components of BTB

The BTB exhibits several unique and notable ultrastructural characteristics, particularly characterized by densely arranged bundles of actin filaments that are oriented perpendicularly to the plasma membrane. These actin bundles are interposed between the cisternae of the endoplasmic reticulum and the plasma membranes of neighboring Sertoli cells, referred to as the basal ectoplasmic specialization (ES). Such ultrastructural elements are consistently observed on both sides of adjacent Sertoli cells, highlighting the intricate organization and functional significance of the BTB in maintaining testicular integrity and spermatogenesis [11,12].

**Figure 1:**
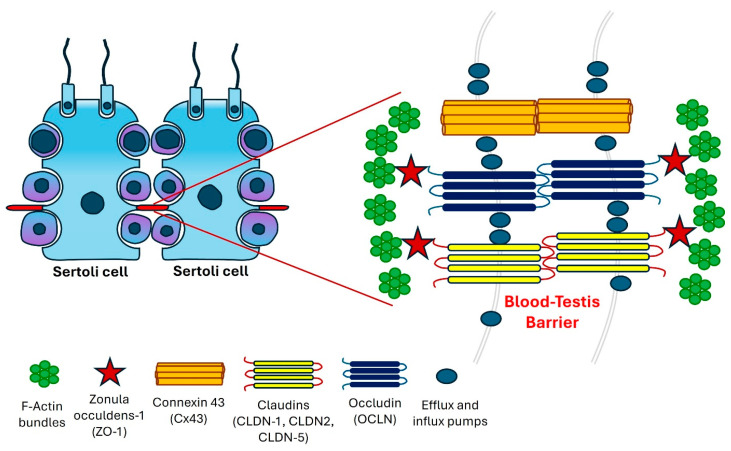
A graphical representation of the blood–testis barrier (BTB) located within the seminiferous epithelium.

Sertoli cells play a crucial role in shielding auto-immunogenic germ cells from the immune response of the host by forming the BTB/Sertoli cell barrier [13]. Unlike other tissue barriers, the BTB is distinguished by its unique composition of four different types of cell junctions. It is primarily constructed from tight junctions (TJs) between capillary endothelial cells, with regulatory contributions from the basal lamina, pericytes, astrocytes, and neurons [14]. It represents one of the most robust junctional structures in mammals, characterized by TJs, gap junctions (GJs), ectoplasmic specializations, and desmosomes among Sertoli cells (Figure 1). Among these junctions, tight junctions are particularly crucial, fulfilling both gate and fence functions, thereby constituting the most essential component of the BTB [6].

TJs in the testis operate alongside ectoplasmic specializations, performing complementary roles. Desmosomes serve as cell–cell junctions that facilitate strong adhesion, while GJs function as channels enabling the diffusion of metabolites, second messengers, ions, and other small molecules under 1 kDa [15,16]. TJs along with ectoplasmic specializations and GJs are associated with actin microfilaments in contrast to desmosomes, which are linked to intermediate filaments [6,17].

These junctions create paired strands between neighboring cells, providing mechanical stability and regulating the passage of small molecules and ions through the paracellular space. In addition to their structural role, TJ proteins are involved in maintaining cellular polarity and facilitating paracellular transport while also recruiting signaling proteins that participate in diverse cellular activities. Disruptions in the architecture and function of TJs can lead to a variety of diseases. 

The functionality of TJs in Sertoli cells declines with age, closely correlating with age-related testicular dysfunction [18]. These junctions create paired strands between neighboring cells, providing mechanical stability and regulating the passage of small molecules and ions through the paracellular space. In addition to their structural role, TJ proteins are involved in maintaining cellular polarity and facilitating paracellular transport while also recruiting signaling proteins that participate in diverse cellular activities. Disruptions in the architecture and function of TJs can lead to a variety of diseases [19]. TJs and ectoplasmic specializations exhibit high vulnerability to damage from environmental toxins and thermal stress [20]. Elevated temperatures in the scrotal region can lead to germ cell apoptosis, resulting in conditions such as oligospermia or azoospermia across various species [21]. This phenomenon is accompanied by reversible alterations in the concentrations of proteins associated with TJs and ectoplasmic specializations [22]. Additionally, the integrity of the BTB is compromised, indicating that these modifications are, in part, attributable to the distinctive arrangement of cellular junctions within this barrier [6]. 

Tight junctions, which originate from epithelial cells, are complex membrane structures characterized by the presence of various integral membrane proteins, including claudins, zonula occludens-1, and occludin [14]. These integral proteins form the foundational framework of TJs, organized as discrete particles along junctional strands, contributing minimally to the overall intramembranous particle composition at the junctional fibrils. The architecture of TJs features a transmembrane region where identical transmembrane proteins are anchored on both sides [23]. On the cytoplasmic side, scaffolding proteins connect to the actin cytoskeleton, playing crucial roles in signaling pathways and the regulation of junctional structure and function [9]. Claudins and occludin, key components of cell junctions, provide structural integrity to tight junctions, with members of the claudin family exhibiting diverse functions, including barrier formation and the regulation of small-molecule and ion permeability [19].

Claudins plays a crucial role in the structure and functionality of tight junctions, characterized by four membrane-spanning segments that consist of two extracellular loops along with N- and C-terminal cytoplasmic domains. The extracellular loops exhibit a high degree of conservation, while the C-terminal domain is essential for the proper localization of claudins within TJs. Claudin proteins are responsible for the formation of TJ fibrils [24]. As part of a multigene family, approximately 27 claudins exhibit distinct patterns of expression that are specific to various tissues, with a molecular weight ranging from 20 to 34 kDa [19]. Claudins are crucial in modulating transepithelial permeability by controlling the paracellular passage of small molecules and ions across the epithelium [19]. 

The claudin family in mammals comprises a minimum of 24 distinct members. Based on sequence similarities, they are classified into classical and nonclassical types. Claudins 1–10, 14, 15, 17, and 19 are classified as classical, whereas 11–13, 16, 18, and 20–24 are nonclassical types [6]. Most cells express multiple claudins, which collectively influence the paracellular electrical resistance and charge selectivity of TJs. This indicates that a claudin molecule can interact with another claudin, whether of the same or different type, on the neighboring cell membrane [24].

Claudin-1 (CLDN-1), a protein with a molecular weight of 22 kDa, is the inaugural member of the claudin family that has been discovered and exhibits high expression levels in various organs, including the intestine, spleen, brain, liver, kidney, and testis [25]. *CLDN-1* is involved in various signal transduction pathways, including those related to gene expression, polarization, proliferation, and differentiation [26]. The mice lacking *Cldn–1* exhibited mortality within one day post-birth and demonstrated significant impairments in the epidermal permeability barrier [27]. Individuals with a deficiency of *CLDN-1* experience neonatal ichthyosis–sclerosing cholangitis (NISCH) syndrome, characterized by a skin phenotype resembling ichthyosis [28].

Claudin-2 is a tight junction protein that is encoded by the *CLDN-2* gene, selectively forms channels for cations, and is predominantly found in leaky epithelial tissues. Its mRNA is particularly abundant in the kidneys, specifically localized to the proximal tubules, and in the gastrointestinal system, with the highest levels detected in the small intestine, liver, gall bladder, and pancreas [29]. This protein facilitates the formation of paracellular channels and is believed to aid in the transport of water and sodium, balancing the chloride and bicarbonate actively secreted by pancreatic duct cells via cystic fibrosis transmembrane conductance protein [30]. The expression of claudin-2 is subject to dynamic regulation, with notable upregulation occurring during inflammatory responses [31]. *CLDN-2* has been recognized as a direct target of the vitamin D receptor, which is implicated in various aspects of the pathogenesis of inflammatory bowel diseases [32]. 

Claudin-11 (CLDN11) is an important transmembrane protein that plays a significant role in the formation of TJs, categorizing it as a key component within the BTB family of TJ proteins [33]. CLDN11 is specifically found at the TJ of the central nervous system myelin and in the inner ear and the testis [34], and these proteins are known to form homophilic interactions [35]. In mice lacking claudin-11, the BTB is compromised, resulting in an impaired differentiation of early spermatocytes and subsequent cell detachment and apoptosis [33]. Notably, CLDN11 is the only critical claudin protein required for spermatogenesis; its deletion in knockout mice results in infertility, while the absence of claudin-1, -3, -12, or -13 does not affect fertility [34,35]. Apart from male infertility, defects in the *CLDN-11* gene are implicated in several types of cancers [36,37] and hearing loss [38,39]. 

Zonula occludens-1 (ZO-1) functions as an adaptor protein, connecting transmembrane proteins to the actin cytoskeleton. ZO-1, a protein with a molecular weight of 225 kDa, is an integral membrane component located in the outermost layer of TJs [40]. Numerous studies have thoroughly characterized the localization of the scaffold cytoplasmic protein ZO-1, which has been identified at the apical membrane of Sertoli cells where it interacts with mature spermatids within the BTB [9]. ZO-1 (TJ protein 1, TJP1), ZO-2 (TJP2), and ZO-3 (TJP3) are extensively studied adaptor proteins that facilitate the connection of integral membrane TJ proteins, including occludin and claudin, to the actin cytoskeleton. In the testis, ZO-1 is found to coimmunoprecipitate and colocalize with connexin 43, playing a vital role in the regulation of gap junctional communication [6].

Occludin (OCLN), a 65 kDa integral membrane protein, was the first identified protein associated with TJs across various epithelial types [40]. In the testes of adult mice and rats, occludin exhibits a linear distribution within the basal areas of the TJ strands formed by Sertoli cells. The distribution of OCLN coincides with that of ZO-1 in the basal third of Sertoli cells [9]. Occludin is characterized by four membrane-spanning domains, two extracellular loops, and two intracellular segments, and it is expressed in various cell types and organs, such as the brain and liver [6]. In patients with nonobstructive azoospermia, the presence of claudin-11, occludin, and ZO-1 correlates with increased apoptosis and the development of irregular or unstained tight junctions [41].

Connexin 43 (Cx43) serves as the primary protein within the GJs of the testis, suggesting its potential significance in testicular development and subsequent spermatogenesis [9]. In fetal and neonatal rats, as spermatogenesis begins, Cx43 is observed in the intercellular spaces among Leydig cells, Sertoli cells, and between Sertoli cells and germ cells [42]. Cx43 knockout mice exhibit neonatal mortality, attributed to the essential role of Cx43 in the development of the heart and brain [43]. Throughout testis development, Cx43 found in the perinatal testis regulates Sertoli cell differentiation and sustains the population of germ cells, including gonocytes and primitive spermatogonia, via Cx43-mediated gap junctions connecting germ cells with neighboring supporting cells [42]. Beyond the regulatory role of Cx43 GJ channels, substantial evidence indicates that Cx43 also influences spermatogenesis by modulating tight and anchoring junctions, which are intricately associated with the BTB, essential for establishing and maintaining Sertoli cell polarity [42].

Apart from these, several other proteins play a crucial role in the integrity of the BTB and cell adhesion and migration. These include membrane proteins: junctional adhesion molecule (JAM), coxsackie and adenovirus receptor (CAR), Cx-33, Cx-45, Cx-57, and N-Cadherin; adaptor proteins: vinculin, α-catenin, β-catenin, and γ-catenin; and a scaffolding protein: actin. All of these proteins are involved in the ultrastructure and permeability of the BTB [6,44,45]. 

Abnormalities in these proteins can result in the dysfunction of the BTB, potentially triggering an immune response directed at meiotic and postmeiotic cells, which may ultimately culminate in spermatogenic failure and male infertility. Furthermore, the role of the BTB may be impaired by genetic defects that affect the development and functionality of cell junctions [46]. The adhesion protein nectin-like molecule 2 (NECL2) plays a crucial role in spermatogenesis by facilitating the interactions between Sertoli cells and germ cells. A deficiency in Necl2 results in male infertility in mice, characterized by abnormal BTB protein levels, including CLDN3, CLDN11, and Cx43. NECL2 is known to interact and colocalize with other adhesion proteins at the BTB, such as Cx43, Occludin, and N-cadherin. It is essential for regulating the dynamics of the BTB during the passage of preleptotene spermatocytes, and its absence leads to the structural damage of the barrier. Furthermore, the deletion of Necl2 significantly disrupts the testicular transcriptome, particularly affecting the expression of genes associated with spermatogenesis [47].

Infertility is one of the primary complex disorders with a spectrum of phenotypes. It occurs as either syndromic or non-syndromic [48]. Male factors contribute to 50% of infertility cases reported, with a prevalence of 11.4% among men aged between 15 and 49 years [49]. There are several factors associated with male infertility including hormonal defects, chronic health issues, and environmental and lifestyle changes [50]. In addition, around 30% of male infertility cases are attributed to genetic causes, while 25–30% are classified as idiopathic, potentially associated with unidentified genetic abnormalities [48]. A primary factor contributing to unexplained male infertility is the disruption of endocrine function during testicular development in the neonatal phase, which can be influenced by environmental toxins as well as genetic and epigenetic factors [51]. These influences are associated with conditions such as testicular dysgenesis, infertility, and testicular cancer. It is posited that these various factors may affect the regulation of the BTB, thereby playing a role in the etiopathology of male infertility [46]. Research indicates that certain drugs capable of mitigating BTB damage caused by cadmium in rat testes have the potential to alleviate BTB injury and could serve as promising treatments for male infertility [52].

In certain instances of male infertility, including varicocele and cryptorchidism, alterations in the BTB and the expression of its associated proteins are believed to contribute to impaired spermatogenesis. Research indicates that in a rat model of varicocele, there is a significant downregulation of CLDN-11, E-cadherin, and α-catenin, which compromises the structural integrity of the BTB [53,54]. Furthermore, undescended testes exhibit a loss of normal BTB functionality and reduced spermatogenesis, implying that an abnormal organization of CLDN-11 may be a factor in male infertility linked to undescended testes [33]. Additionally, lifestyle factors such as the consumption of a high-fat diet, which leads to obesity, chronic stress, and insufficient sleep can negatively affect sperm quality by damaging the BTB [52].

The BTB is adversely affected by a diet high in cholesterol, which significantly and progressively increases lipid accumulation within the seminiferous tubules [55]. Studies show that chronic stress negatively influences essential proteins and sperm attributes associated with the BTB, notably compromising the integrity of the BTB and ZO-1, along with decreasing CLDN-11 levels [56]. Furthermore, sleep deprivation has been found to diminish the expression of TJ proteins, actin, and androgen receptors, leading to reduced sperm viability and motility due to alterations in the blood–testis environment [57].

The BTB is essential in safeguarding luminal germ cells from exposure to the circulatory and lymphatic systems, thereby creating an immune-privileged environment conducive to the completion of meiosis, particularly when coupled with localized immunosuppression. Infections and inflammatory processes within the reproductive system can adversely affect male fertility. BTB is essential for shielding haploid germ cells from immune attacks, with IL-6 potentially influencing the downregulation of occludin expression and alterations in BTB permeability observed in autoimmune orchitis models [58]. Additionally, IL-17A facilitates the recruitment of immune cells to the testicular interstitium while simultaneously compromising BTB functionality [59]. IL-6 disrupts the structural integrity of the BTB, leading to changes in the localization and steady-state levels of integral membrane proteins associated with the BTB [60]. Spermatogenesis predominantly occurs within the testis [52].

### Toxicants Affecting BTB Function

Various environmental endocrine-disrupting compounds (EDCs), synthetic substances that interfere with hormonal functions in humans and animals, are present in the environment due to industrial and manufacturing processes [61]. These toxicants, such as cadmium, phthalates, perfluorooctane sulfonate, and bisphenol A (BPA), represent a significant emerging factor associated with decreased sperm counts and male infertility. In vitro models were employed to investigate the molecular mechanisms through which toxicants cause a dysfunction in male reproductive health [62,63]. Human exposure to BPA primarily occurs via contaminated food and water; however, it may also migrate from polycarbonate or plastic containers and bottles, particularly when subjected to higher temperatures [64]. BPA interacts with estrogen and androgen receptors in Sertoli cells, disrupting their functionality and negatively impacting male reproductive health [65,66]. Furthermore, BPA downregulates the expression of the Cx43 gene, leading to a redistribution of Cx43 at cell junctions and an increase in the pore size of gap junctions [67].

Cadmium, a heavy metal recognized as an endocrine disruptor, has been shown to induce male reproductive toxicity in both humans and rodents [62]. Heavy industries such as metal mining and refining, battery manufacturing, and fertilizer production emit cadmium into the atmosphere, subsequently infiltrating the food chain. Further, cigarette smoking serves as another significant source of cadmium exposure for humans [68,69]. Cadmium alters various hormone levels within the hypothalamic–pituitary–testicular axis, including testosterone, LH, and FSH. It has also been shown to have a detrimental impact on Leydig cell steroidogenesis [61]. The primary target of cadmium exposure is the BTB, which causes the defragmentation of the Sertoli cells’ actin filaments and TJ fibrils. It downregulates the expression of FAK, a nonreceptor tyrosine kinase protein, which interacts with TJ proteins OCLN and ZO-1 [70]. This results in the disturbance of the protein complex within Sertoli cells, making the BTB insensitive to cadmium toxicity [61].

Fine particulate matter exposure is linked to male reproductive toxicity through the degradation of blood–testis barrier proteins [71]. Similarly, micro and nano plastics are prevalent in human environments and can infiltrate the body via water, the food chain, and inhalation. Research indicates that these particles can compromise the BTB in rodent models by significantly reducing the levels of TJ proteins [72]. Increased concentrations of urea and uric acid in seminal plasma negatively affect fertilization rates, suggesting their possible involvement as factors contributing to reduced fertility [73].

All these environmental toxicants can provoke hormonal imbalances, initiate apoptosis, and impede the proliferation of spermatogenic cells by adversely affecting the structure and functionality of Sertoli cells, leading to the impairment of the BTB and an increase in reactive oxygen species (ROS) production [73,74,75]. The associated health risks may be mitigated by the oral intake of certain bioactive compounds. Furthermore, antioxidant medications have been shown to alleviate oxidative stress-related damage to the BTB, thereby supporting male fertility. Evidence from animal studies suggests that antioxidant supplementation could serve as a promising therapeutic approach for both preventing and alleviating BTB damage [52]. On the other hand, exposure to heavy metals such as copper and nickel showed an increase in L-serine and glucose [73]. Serine serves as a precursor for glycine and cysteine and is involved in the synthesis of glutathione. The production of glutathione plays a crucial role in reducing oxidative stress and enhancing overall activity, thereby boosting the function of antioxidant enzymes [76]. Similarly, L-cysteine serves as a potent antioxidant and may offer a viable therapeutic approach to mitigate the risk of various diseases [77].

## 4. L-Cysteine

L-cysteine (L-Cys) is classified as a non-essential amino acid, serving as a fundamental component in protein synthesis [78]. L-cysteine is the amino acid responsible for forming disulfide bonds, which are covalent connections essential for the proper folding and stabilization of protein tertiary structures, thus facilitating their biological functions [79]. Cysteine can be incorporated via various metabolic pathways based on cellular requirements, leading to the formation of sulfur-containing compounds [80]. L-cysteine is a compound widely utilized in the pharmaceutical development of various medications. Nevertheless, the number of clinical trials investigating the effects of L-cysteine on human health and well-being is limited. Consequently, the implications of L-cysteine consumption, whether through pharmaceuticals or dietary supplements, remain a contentious topic [77].

Various derivatives of L-cysteine function as antioxidants, playing a crucial role in preserving redox balance. Cysteine persulfide, a derivative of L-cysteine, features an extra sulfur atom attached to a cysteinyl thiol group, functioning as a reactive sulfur species that plays a crucial role in maintaining redox homeostasis within cells [81]. L-cysteine serves as a precursor in the synthesis of glutathione, a crucial antioxidant. The reduced variant of glutathione is essential for protecting the body against damage caused by oxidative stress. This characteristic stems from its capacity to neutralize reactive species that may harm cells and tissues [77]. Consequently, supplementing the diet with L-cysteine reinstates glutathione synthesis when it has been impaired, thereby enhancing redox equilibrium and mitigating oxidative stress [82]. The removal of free radicals may also confer advantages, such as shorter recovery times after specific surgical interventions [83]. Furthermore, the antioxidant function of L-cysteine is linked to a decreased likelihood of chronic obstructive pulmonary disease [84], acute ischemic stroke [85], and a lower incidence of noise-induced hearing loss [86].

The immune system’s activity is subject to regulation, with evidence indicating that L-cysteine influences the production of effector molecules, notably IL-17 [87]. This cytokine, primarily synthesized by Th17 cells, interacts with epithelial cells, fibroblasts, and various immune cells [88]. Furthermore, research has shown that NAC administration can significantly enhance transplant-free survival rates in patients experiencing acute liver failure unrelated to acetaminophen, particularly when given in the early phases of hepatic encephalopathy. This beneficial outcome is attributed to the modulation of IL-17 production, which plays a vital role in the advancement of encephalopathy [87]. NAC exhibits potent antitumoral activity [89]. NAC has been documented to mitigate the toxic effects of heavy metals like cadmium and lead while also protecting against environmental pollutants [90]. Several studies indicate that certain amino acids, including L-cysteine, may confer cardiovascular advantages, such as decreased arterial stiffness and lower blood pressure, which could mitigate some risk factors associated with vascular events in healthy women [91].

## 5. Protective Effect of L-Cysteine in Testicular Cells

N-acetyl-L-cysteine (NAC), an amino acid derivative of L-cysteine, exhibits notable protective properties against male reproductive dysfunction induced by busulfan, likely via alterations in the Nrf2/HO-1 signaling pathway [92]. Additionally, NAC has been shown to mitigate chromium-induced oxidative damage in the testes of mice [93] and to decrease lipid peroxidation and 8-hydroxy-2-deoxyguanosine formation in the testes of rats exposed to sodium fluoride [94]. Combined supplementation with α-lipoic acid and NAC mitigated the testicular spermatogenic and steroidogenic impairments caused by reduced ROS generation during intensive swimming [95].

NAC attenuates the BTB damage caused by the synchrotron radiation X-ray [96]. NAC may be used as a preventative measure against iron overload-induced testicular damage [97]. The antioxidant properties of NAC mitigate the harm inflicted on testicular cells by a range of chemicals and radiation [98,99,100,101]. NAC is an effective mucolytic agent that alleviates excessive mucus production and boosts the activity of glutathione S-transferase. As a potent antioxidant, it presents a promising therapeutic avenue for conditions associated with oxidative stress and the production of free oxygen radicals. The significant antioxidant capacity of NAC stems from its function as a precursor to glutathione, a key naturally occurring antioxidant in the body [102]. The consumption of dietary antioxidants may positively influence sperm quality and related parameters [103]. Table 1 summarizes the animal studies highlighting the protective effect of NAC against BTB damage and male reproductive toxicity caused by various chemicals and radiations.

L-cysteine treatment increased the expression of testosterone regulatory and BTB genes in human Leydig cells. In addition, L-cysteine increased the levels of testosterone post–treatment [105]. L-cysteine supplementation thus protects the testis from various harmful effects and increases the quality of sperm. BTB disruption caused by oxidative stress can be mitigated by supplementation with L-cysteine and may provide advantageous effects against oxidative stress and impede the infiltration of cytotoxic drugs into the seminiferous tubules via the BTB. There are only limited studies that have shown the beneficial effects of L-cysteine treatment among infertile men (Table 2). 

**Figure 2:**
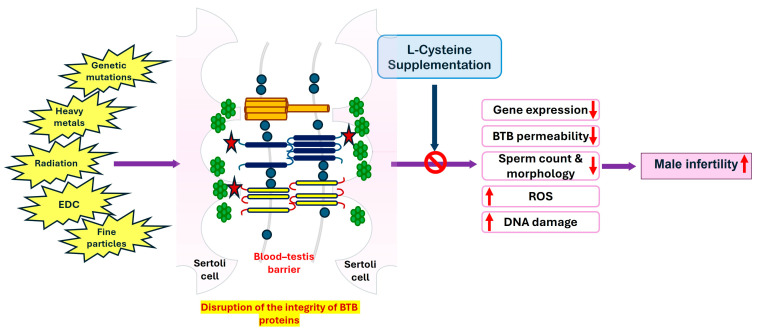
The beneficial effect of L-cysteine supplementation against the damages caused by various external and genetic factors affecting the integrity of BTB proteins leads to reduced permeability and gene expression. EDC, endocrine-disrupting compounds; ROS, reactive oxygen species.

Figure 2 summarizes the beneficial effects of L-cysteine in protecting against the harmful effects of various factors affecting the integrity of the BTB proteins that induce reduced sperm production and increased ROS and DNA damage, leading to male infertility. Clinical trials in infertile men are required to determine whether L-cysteine shows a similar effect by increasing the integrity of the BTB and boosting testosterone production.

## 6. Conclusions

The BTB acts as the Sertoli cell seminiferous epithelium barrier, which is essential for spermatogenesis, providing an immunoprivileged microenvironment for the completion of meiosis. The BTB formed mainly by extensive TJs and GJs among Sertoli cells serves as both an anatomical barrier that restricts the passage of molecules and cells into the adluminal compartment and an immunological barrier that inhibits the intercellular diffusion of substances and molecules. Deficiencies in the proteins responsible for the formation and maintenance of cell junctions may impair the function of the BTB, resulting in male infertility. This review does not encompass the pathways through which these genes interact with other proteins to maintain the integrity of the channels in the Sertoli cells. Oxidative stress is a potent oxidizing agent that can potentially lead to impaired spermatogenesis and male infertility. L-cysteine, an important antioxidant, plays a defensive role against the damage caused by oxidative stress and has demonstrated a protective effect against testicular damage and enhances the expression of BTB genes. Supplementation with L-cysteine may increase the production of testosterone and improve the integrity of the BTB, consequently mitigating the effects of male infertility. 

## Figures and Tables

**Table 1:** Protective role of NAC/L-cysteine against BTB damage and male reproductive toxicity caused by various chemicals and radiations.

Subject Subjects	Sample Size (*n*)	Treatment Dosage Levels	Outcome	Reference
C57BL/6 male mice	32 (*n* = 8/group)	NAC: 10 mg/kg daily Busulfan: 40 mg/kg	NAC has significant protective effects against busulfan-induced male reproductive impairment	[92]
Male Swiss Albino mice	36 (*n* = 6/group)	Cr (IV) exposed: 20 mg Cr/kg NAC pre- and post-treatment: 200 mg/kg Taurine pre- and post-treatment: 1 g/kg	NAC and taurine have the potential to prevent chromium-induced toxicity	[93]
Male Sprague Dawley rats	60 (*n* = 15/group)	Control: saline (10 mL/kg) NaF treated: 25 mg/kg/d NAC: 150 mg/kg/day	NAC acts as an antidote against fluoride-induced male reproductive toxicity	[94]
Male Wistar strain rats	48 (*n* = 12/group)	NAC: 50 mg/100 g BW/day α-lipoic acid: 3 mg/100 g BW/day	The combined supplementation of NAC and α-lipoic acid protected the forced-intensive-swimming-induced male reproductive dysfunctions	[95]
Male Sprague Dawley rats	*n* = 6–8/group	NAC: 25 or 125 mg/kg	NAC (125 mg) attenuated the BTB damage induced by X-rays	[96]
Male albino rats	40 (*n* = 10/group)	NAC: 150 mg /kg Iron dextran: 60 mg/kg/day AOP: 150 mg/kg/day	NAC and AOP protect against iron overload testis damage	[97]
Rats	18 (*n* = 6/group)	NAC: 20 mg/kg	NAC improved the tissue damage in the contralateral testes after torsion	[101]
Male Wistar albino rats	28 (*n* = 7/group)	NAC low dose: 10 mg/kg NAC high dose: 100 mg/kg	High-dose NAC increased Sertoli cell numbers and decreased the loss of testis volume, thus protecting from ischemic damage	[100]
Male mice	30 (*n* = 5/group)	NAC: 150 mg/kg Art: 50 mg/kg	NAC neutralizes the effects of Art (sperm defects, oxidative stress)	[99]
Male Wistar albino rats	32 (*n* = 8/groups)	NAC: 150 mg/kg/day	NAC reduced the undescended testis-related testicular damage by reducing oxidative stress	[98]
Male Wistar albino rats	48 (*n* = 6/groups)	NAC: 300 mg/kg BW Cadmium: 300 ppm Lead: 1000 ppm	NAC encountered the reproductive toxicity of cadmium and lead	[104]

AOP, acetylated peptide; Art, artemisinin; BTB, blood–testis barrier; BW, body weight; Cr, chromium; NAC, N-acetyl-L-Cysteine; NaF, sodium fluoride.

**Table 2:** The beneficial role of NAC/L-cysteine in improving sperm parameters among infertile men.

Subject Subjects	Sample Size (*n*)	Treatment Dosage Levels	Outcome	Reference
Infertile men	35 (C-20, T-15)	NAC: 600 mg/day	NAC improved chromatin integrity	[106]
Idiopathic infertile men	120 (C-60, T-60)	NAC: 600 mg/day	NAC improved the volume, motility, and viscosity of semen	[107]
Infertile men with oligo-asthenoteratospermia	468	Se: 200 µg/day NAC: 600 mg/day	NAC+Se improved semen quality	[108]
Infertile men with asthenoteratozoospermia	50	NAC: 600 mg/day	NAC improved sperm parameters and antioxidant levels	[109]

C, control; NAC, N-acetyl-L-Cysteine; Se, selenium; T, treatment group.
